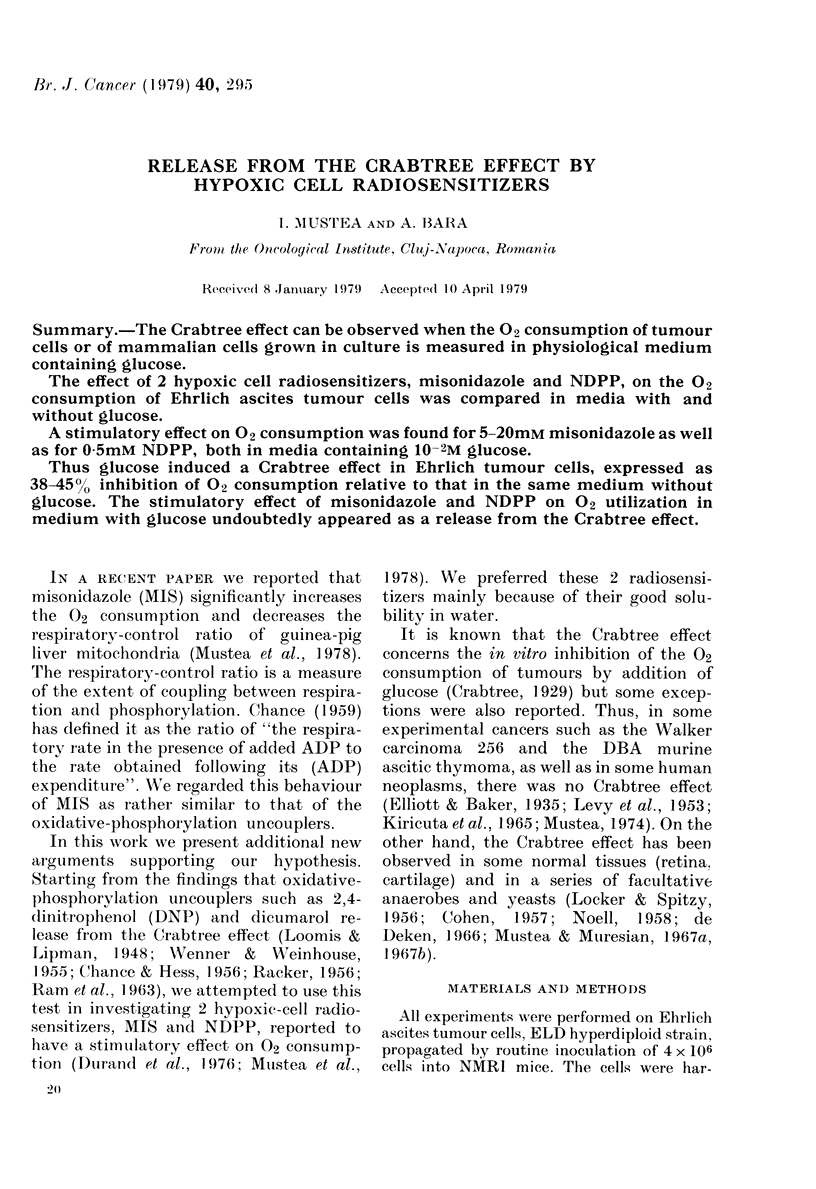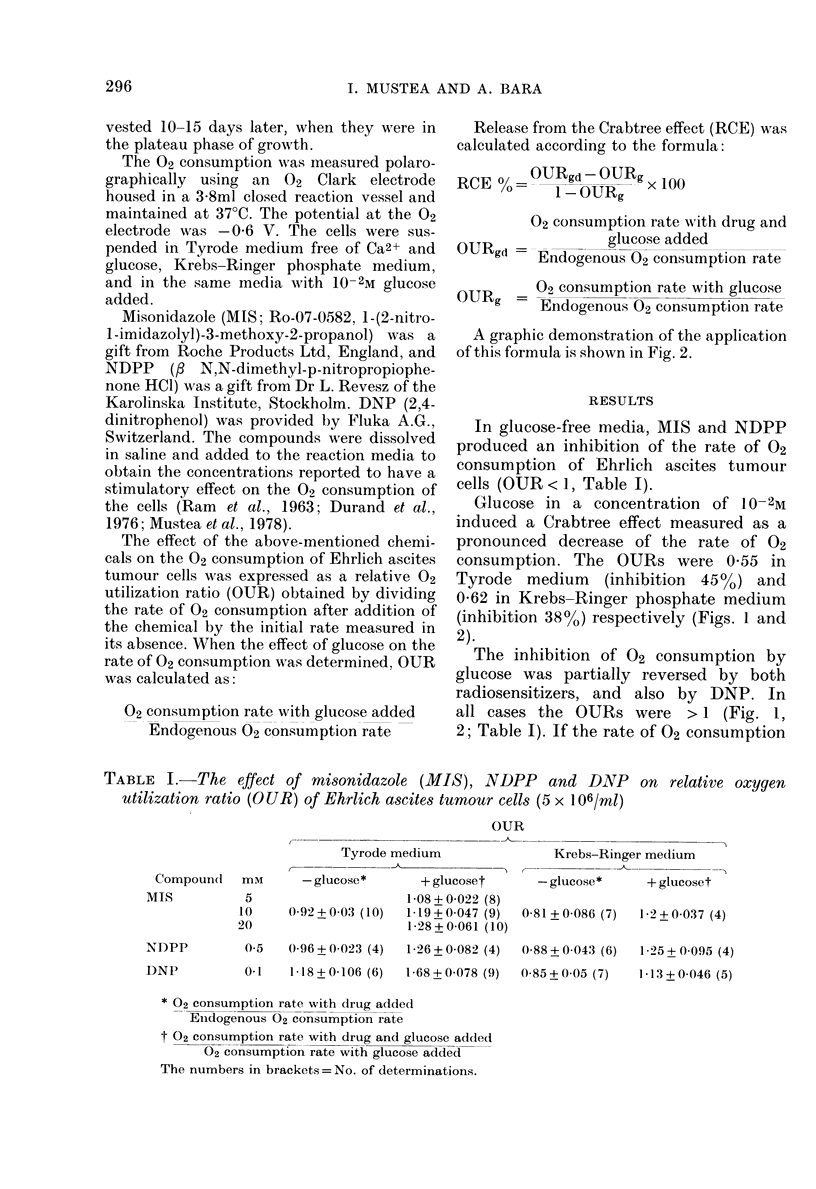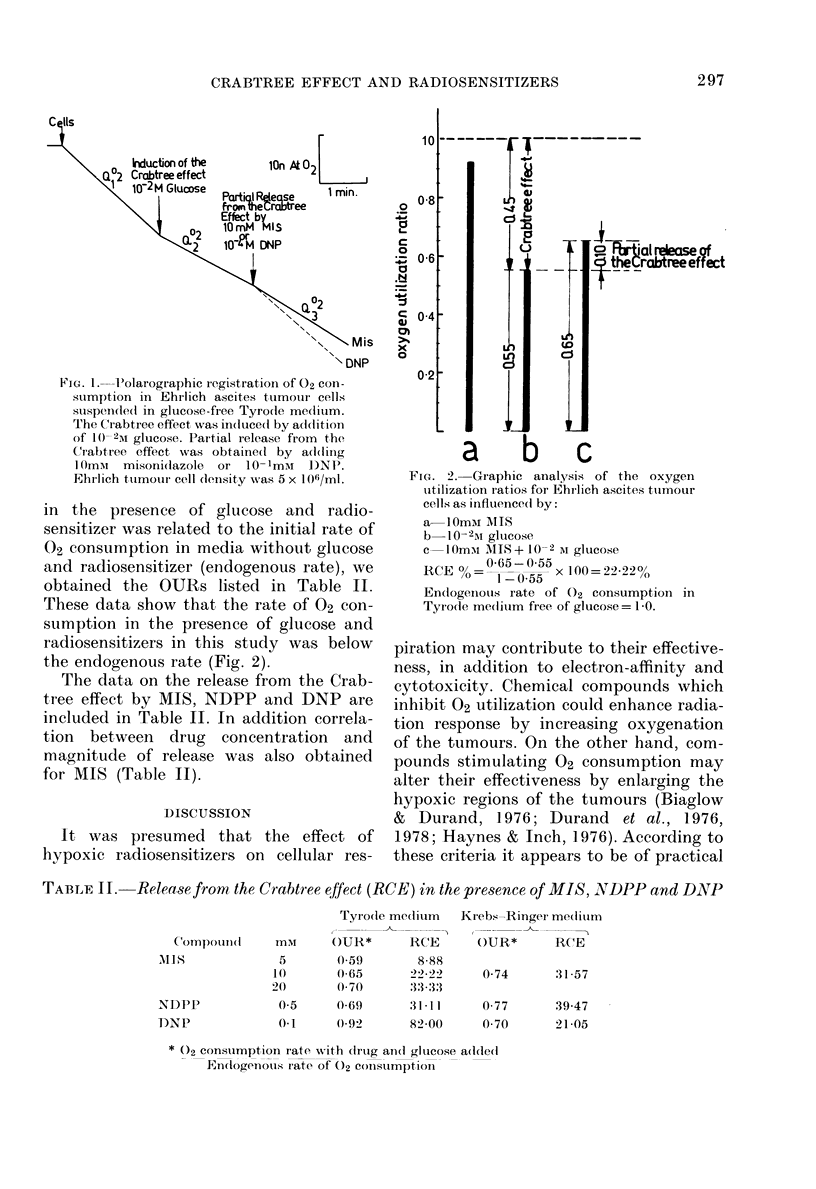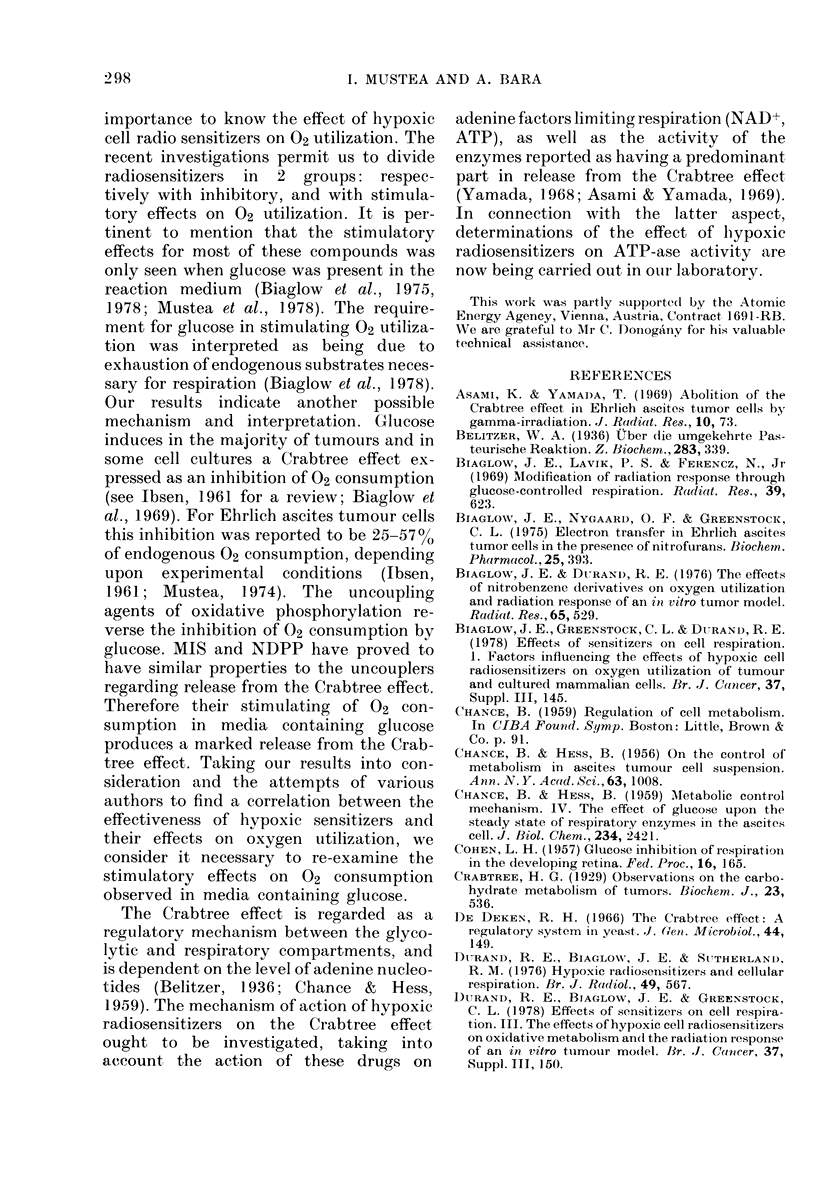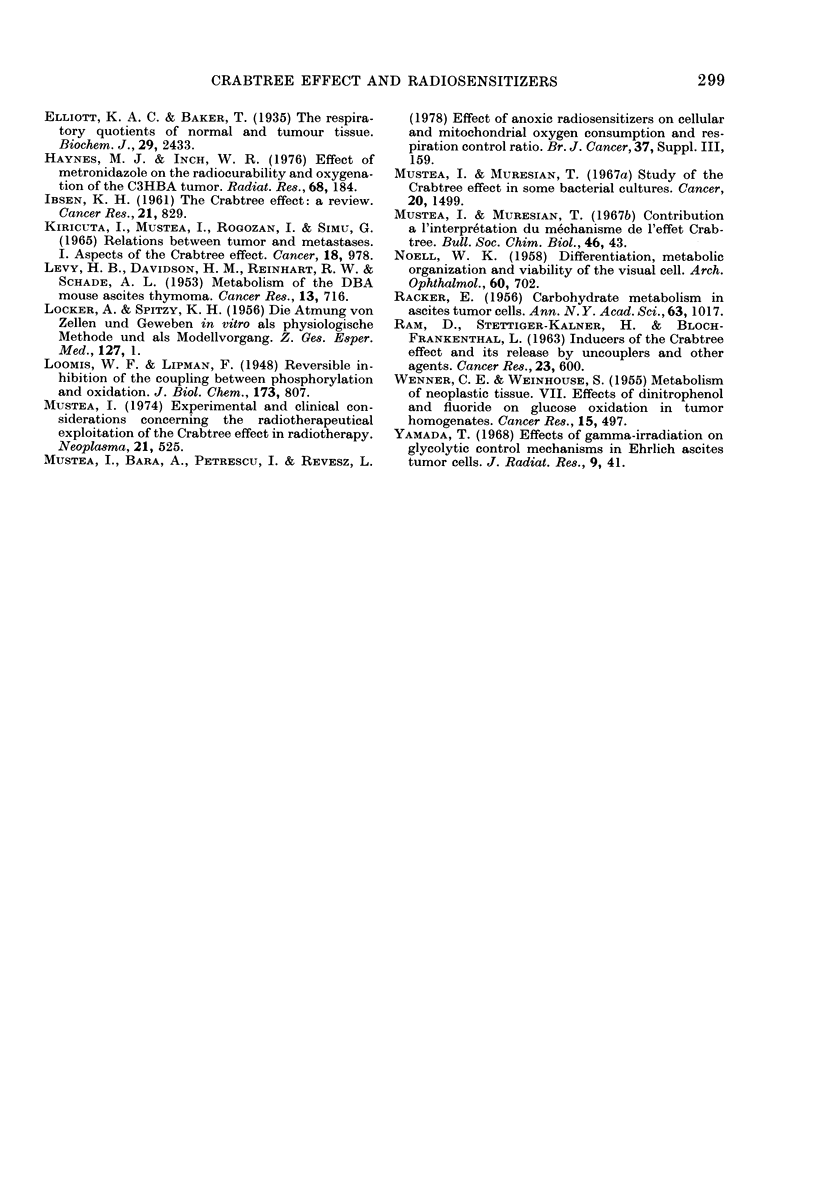# Release from the Crabtree effect by hypoxic cell radiosensitizers.

**DOI:** 10.1038/bjc.1979.178

**Published:** 1979-08

**Authors:** I. Mustea, A. Bara

## Abstract

The Crabtree effect can be observed when the O2 consumption of tumour cells or of mammalian cells grown in culture is measured in physiological medium containing glucose. The effect of 2 hypoxic cell radiosensitizers, misonidazole and NDPP, on the O2 consumption of Ehrlich ascites tumour cells was compared in media with and without glucose. A stimulatory effect on O2 consumption was found for 5--20mM misonidazole as well as for 0.5mM NDPP, both in media containing 10(-2)M glucose. Thus glucose induced a Crabtree effect in Ehrlich tumour cells, expressed as 38--45% inhibition of O2 consumption relative to that in the same medium without glucose. The stimulatory effect of misonidazole and NDPP on O2 utilization in medium with glucose undoubtedly appeared as a release from the Crabtree effect.


*Br. J. Cancer* (1979) **40**, 295

### RELEASE FROM THE CRABTREE EFFECT BY HYPOXIC CELL RADIOSENSITIZERS

I. MUSTEA AND A. BARA

*From the Oncological Institute, Cluj-Napoca, Romania*

Received 8 January 1979 Accepted 10 April 1979

**Summary.—The Crabtree effect can be observed when the $O_2$ consumption of tumour cells or of mammalian cells grown in culture is measured in physiological medium containing glucose.**

**The effect of 2 hypoxic cell radiosensitizers, misonidazole and NDPP, on the $O_2$ consumption of Ehrlich ascites tumour cells was compared in media with and without glucose.**

**A stimulatory effect on $O_2$ consumption was found for 5–20mM misonidazole as well as for 0·5mM NDPP, both in media containing $10^{-2}$M glucose.**

**Thus glucose induced a Crabtree effect in Ehrlich tumour cells, expressed as 38–45% inhibition of $O_2$ consumption relative to that in the same medium without glucose. The stimulatory effect of misonidazole and NDPP on $O_2$ utilization in medium with glucose undoubtedly appeared as a release from the Crabtree effect.**

IN A RECENT PAPER we reported that misonidazole (MIS) significantly increases the $O_2$ consumption and decreases the respiratory-control ratio of guinea-pig liver mitochondria (Mustea *et al.*, 1978). The respiratory-control ratio is a measure of the extent of coupling between respiration and phosphorylation. Chance (1959) has defined it as the ratio of "the respiratory rate in the presence of added ADP to the rate obtained following its (ADP) expenditure". We regarded this behaviour of MIS as rather similar to that of the oxidative-phosphorylation uncouplers.

In this work we present additional new arguments supporting our hypothesis. Starting from the findings that oxidative-phosphorylation uncouplers such as 2,4-dinitrophenol (DNP) and dicumarol release from the Crabtree effect (Loomis & Lipman, 1948; Wenner & Weinhouse, 1955; Chance & Hess, 1956; Racker, 1956; Ram *et al.*, 1963), we attempted to use this test in investigating 2 hypoxic-cell radiosensitizers, MIS and NDPP, reported to have a stimulatory effect on $O_2$ consumption (Durand *et al.*, 1976; Mustea *et al.*, 1978). We preferred these 2 radiosensitizers mainly because of their good solubility in water.

It is known that the Crabtree effect concerns the *in vitro* inhibition of the $O_2$ consumption of tumours by addition of glucose (Crabtree, 1929) but some exceptions were also reported. Thus, in some experimental cancers such as the Walker carcinoma 256 and the DBA murine ascitic thymoma, as well as in some human neoplasms, there was no Crabtree effect (Elliott & Baker, 1935; Levy *et al.*, 1953; Kiricuta *et al.*, 1965; Mustea, 1974). On the other hand, the Crabtree effect has been observed in some normal tissues (retina, cartilage) and in a series of facultative anaerobes and yeasts (Locker & Spitzy, 1956; Cohen, 1957; Noell, 1958; de Deken, 1966; Mustea & Muresian, 1967*a*, 1967*b*).

### MATERIALS AND METHODS

All experiments were performed on Ehrlich ascites tumour cells, ELD hyperdiploid strain, propagated by routine inoculation of $4 \times 10^6$ cells into NMRI mice. The cells were harvested 10–15 days later, when they were in the plateau phase of growth.

The $O_2$ consumption was measured polarographically using an $O_2$ Clark electrode housed in a 3·8ml closed reaction vessel and maintained at 37°C. The potential at the $O_2$ electrode was $-0{\cdot}6$ V. The cells were suspended in Tyrode medium free of $Ca^{2+}$ and glucose, Krebs–Ringer phosphate medium, and in the same media with $10^{-2}$M glucose added.

Misonidazole (MIS; Ro-07-0582, 1-(2-nitro-1-imidazolyl)-3-methoxy-2-propanol) was a gift from Roche Products Ltd, England, and NDPP (β N,N-dimethyl-p-nitropropiophenone HCl) was a gift from Dr L. Revesz of the Karolinska Institute, Stockholm. DNP (2,4-dinitrophenol) was provided by Fluka A.G., Switzerland. The compounds were dissolved in saline and added to the reaction media to obtain the concentrations reported to have a stimulatory effect on the $O_2$ consumption of the cells (Ram *et al.*, 1963; Durand *et al.*, 1976; Mustea *et al.*, 1978).

The effect of the above-mentioned chemicals on the $O_2$ consumption of Ehrlich ascites tumour cells was expressed as a relative $O_2$ utilization ratio (OUR) obtained by dividing the rate of $O_2$ consumption after addition of the chemical by the initial rate measured in its absence. When the effect of glucose on the rate of $O_2$ consumption was determined, OUR was calculated as:

$$\frac{O_2 \text{ consumption rate with glucose added}}{\text{Endogenous } O_2 \text{ consumption rate}}$$

Release from the Crabtree effect (RCE) was calculated according to the formula:

$$\text{RCE } \% = \frac{OUR_{gd} - OUR_g}{1 - OUR_g} \times 100$$

$$OUR_{gd} = \frac{O_2 \text{ consumption rate with drug and glucose added}}{\text{Endogenous } O_2 \text{ consumption rate}}$$

$$OUR_g = \frac{O_2 \text{ consumption rate with glucose}}{\text{Endogenous } O_2 \text{ consumption rate}}$$

A graphic demonstration of the application of this formula is shown in Fig. 2.

### RESULTS

In glucose-free media, MIS and NDPP produced an inhibition of the rate of $O_2$ consumption of Ehrlich ascites tumour cells (OUR < 1, Table I).

Glucose in a concentration of $10^{-2}$M induced a Crabtree effect measured as a pronounced decrease of the rate of $O_2$ consumption. The OURs were 0·55 in Tyrode medium (inhibition 45%) and 0·62 in Krebs–Ringer phosphate medium (inhibition 38%) respectively (Figs. 1 and 2).

The inhibition of $O_2$ consumption by glucose was partially reversed by both radiosensitizers, and also by DNP. In all cases the OURs were > 1 (Fig. 1, 2; Table I). If the rate of $O_2$ consumption

TABLE I.—*The effect of misonidazole (MIS), NDPP and DNP on relative oxygen utilization ratio (OUR) of Ehrlich ascites tumour cells* (5 × 10⁶/*ml*)

| | | OUR | | | |
|---|---|---|---|---|---|
| | | Tyrode medium | | Krebs–Ringer medium | |
| Compound | mM | −glucose* | +glucose† | −glucose* | +glucose† |
| MIS | 5 | | 1·08 ± 0·022 (8) | | |
| | 10 | 0·92 ± 0·03 (10) | 1·19 ± 0·047 (9) | 0·81 ± 0·086 (7) | 1·2 ± 0·037 (4) |
| | 20 | | 1·28 ± 0·061 (10) | | |
| NDPP | 0·5 | 0·96 ± 0·023 (4) | 1·26 ± 0·082 (4) | 0·88 ± 0·043 (6) | 1·25 ± 0·095 (4) |
| DNP | 0·1 | 1·18 ± 0·106 (6) | 1·68 ± 0·078 (9) | 0·85 ± 0·05 (7) | 1·13 ± 0·046 (5) |

* $\frac{O_2 \text{ consumption rate with drug added}}{\text{Endogenous } O_2 \text{ consumption rate}}$

† $\frac{O_2 \text{ consumption rate with drug and glucose added}}{O_2 \text{ consumption rate with glucose added}}$

The numbers in brackets = No. of determinations.

FIG. 1.—Polarographic registration of $O_2$ consumption in Ehrlich ascites tumour cells suspended in glucose-free Tyrode medium. The Crabtree effect was induced by addition of $10^{-2}$M glucose. Partial release from the Crabtree effect was obtained by adding 10mM misonidazole or $10^{-1}$mM DNP. Ehrlich tumour cell density was $5 \times 10^6$/ml.

FIG. 2.—Graphic analysis of the oxygen utilization ratios for Ehrlich ascites tumour cells as influenced by:

a—10mM MIS

b—$10^{-2}$M glucose c—10mM MIS + $10^{-2}$ M glucose

$$\text{RCE } \% = \frac{0{\cdot}65 - 0{\cdot}55}{1 - 0{\cdot}55} \times 100 = 22{\cdot}22\%$$

Endogenous rate of $O_2$ consumption in Tyrode medium free of glucose = 1·0.

in the presence of glucose and radiosensitizer was related to the initial rate of $O_2$ consumption in media without glucose and radiosensitizer (endogenous rate), we obtained the OURs listed in Table II. These data show that the rate of $O_2$ consumption in the presence of glucose and radiosensitizers in this study was below the endogenous rate (Fig. 2).

The data on the release from the Crabtree effect by MIS, NDPP and DNP are included in Table II. In addition correlation between drug concentration and magnitude of release was also obtained for MIS (Table II).

### DISCUSSION

It was presumed that the effect of hypoxic radiosensitizers on cellular respiration may contribute to their effectiveness, in addition to electron-affinity and cytotoxicity. Chemical compounds which inhibit $O_2$ utilization could enhance radiation response by increasing oxygenation of the tumours. On the other hand, compounds stimulating $O_2$ consumption may alter their effectiveness by enlarging the hypoxic regions of the tumours (Biaglow & Durand, 1976; Durand *et al.*, 1976, 1978; Haynes & Inch, 1976). According to these criteria it appears to be of practical

TABLE II.—*Release from the Crabtree effect (RCE) in the presence of MIS, NDPP and DNP*

| | | Tyrode medium | | Krebs–Ringer medium | |
|---|---|---|---|---|---|
| Compound | mM | OUR* | RCE | OUR* | RCE |
| MIS | 5 | 0·59 | 8·88 | | |
| | 10 | 0·65 | 22·22 | 0·74 | 31·57 |
| | 20 | 0·70 | 33·33 | | |
| NDPP | 0·5 | 0·69 | 31·11 | 0·77 | 39·47 |
| DNP | 0·1 | 0·92 | 82·00 | 0·70 | 21·05 |

$$* \ \frac{O_2 \text{ consumption rate with drug and glucose added}}{\text{Endogenous rate of } O_2 \text{ consumption}}$$

importance to know the effect of hypoxic cell radio sensitizers on $O_2$ utilization. The recent investigations permit us to divide radiosensitizers in 2 groups: respectively with inhibitory, and with stimulatory effects on $O_2$ utilization. It is pertinent to mention that the stimulatory effects for most of these compounds was only seen when glucose was present in the reaction medium (Biaglow *et al.*, 1975, 1978; Mustea *et al.*, 1978). The requirement for glucose in stimulating $O_2$ utilization was interpreted as being due to exhaustion of endogenous substrates necessary for respiration (Biaglow *et al.*, 1978). Our results indicate another possible mechanism and interpretation. Glucose induces in the majority of tumours and in some cell cultures a Crabtree effect expressed as an inhibition of $O_2$ consumption (see Ibsen, 1961 for a review; Biaglow *et al.*, 1969). For Ehrlich ascites tumour cells this inhibition was reported to be 25–57% of endogenous $O_2$ consumption, depending upon experimental conditions (Ibsen, 1961; Mustea, 1974). The uncoupling agents of oxidative phosphorylation reverse the inhibition of $O_2$ consumption by glucose. MIS and NDPP have proved to have similar properties to the uncouplers regarding release from the Crabtree effect. Therefore their stimulating of $O_2$ consumption in media containing glucose produces a marked release from the Crabtree effect. Taking our results into consideration and the attempts of various authors to find a correlation between the effectiveness of hypoxic sensitizers and their effects on oxygen utilization, we consider it necessary to re-examine the stimulatory effects on $O_2$ consumption observed in media containing glucose.

The Crabtree effect is regarded as a regulatory mechanism between the glycolytic and respiratory compartments, and is dependent on the level of adenine nucleotides (Belitzer, 1936; Chance & Hess, 1959). The mechanism of action of hypoxic radiosensitizers on the Crabtree effect ought to be investigated, taking into account the action of these drugs on adenine factors limiting respiration ($NAD^+$, ATP), as well as the activity of the enzymes reported as having a predominant part in release from the Crabtree effect (Yamada, 1968; Asami & Yamada, 1969). In connection with the latter aspect, determinations of the effect of hypoxic radiosensitizers on ATP-ase activity are now being carried out in our laboratory.

This work was partly supported by the Atomic Energy Agency, Vienna, Austria, Contract 1691-RB. We are grateful to Mr C. Donogány for his valuable technical assistance.

### REFERENCES

Asami, K. & Yamada, T. (1969) Abolition of the Crabtree effect in Ehrlich ascites tumor cells by gamma-irradiation. *J. Radiat. Res.*, **10**, 73.

Belitzer, W. A. (1936) Über die umgekehrte Pasteurische Reaktion. *Z. Biochem.*, **283**, 339.

Biaglow, J. E., Lavik, P. S. & Ferencz, N., Jr (1969) Modification of radiation response through glucose-controlled respiration. *Radiat. Res.*, **39**, 623.

Biaglow, J. E., Nygaard, O. F. & Greenstock, C. L. (1975) Electron transfer in Ehrlich ascites tumor cells in the presence of nitrofurans. *Biochem. Pharmacol.*, **25**, 393.

Biaglow, J. E. & Durand, R. E. (1976) The effects of nitrobenzene derivatives on oxygen utilization and radiation response of an *in vitro* tumor model. *Radiat. Res.*, **65**, 529.

Biaglow, J. E., Greenstock, C. L. & Durand, R. E. (1978) Effects of sensitizers on cell respiration. 1. Factors influencing the effects of hypoxic cell radiosensitizers on oxygen utilization of tumour and cultured mammalian cells. *Br. J. Cancer*, **37**, Suppl. III, 145.

Chance, B. (1959) Regulation of cell metabolism. In *CIBA Found. Symp.* Boston: Little, Brown & Co. p. 91.

Chance, B. & Hess, B. (1956) On the control of metabolism in ascites tumour cell suspension. *Ann. N.Y. Acad. Sci.*, **63**, 1008.

Chance, B. & Hess, B. (1959) Metabolic control mechanism. IV. The effect of glucose upon the steady state of respiratory enzymes in the ascites cell. *J. Biol. Chem.*, **234**, 2421.

Cohen, L. H. (1957) Glucose inhibition of respiration in the developing retina. *Fed. Proc.*, **16**, 165.

Crabtree, H. G. (1929) Observations on the carbohydrate metabolism of tumors. *Biochem. J.*, **23**, 536.

De Deken, R. H. (1966) The Crabtree effect: A regulatory system in yeast. *J. Gen. Microbiol.*, **44**, 149.

Durand, R. E., Biaglow, J. E. & Sutherland, R. M. (1976) Hypoxic radiosensitizers and cellular respiration. *Br. J. Radiol.*, **49**, 567.

Durand, R. E., Biaglow, J. E. & Greenstock, C. L. (1978) Effects of sensitizers on cell respiration. III. The effects of hypoxic cell radiosensitizers on oxidative metabolism and the radiation response of an *in vitro* tumour model. *Br. J. Cancer*, **37**, Suppl. III, 150.

ELLIOTT, K. A. C. & BAKER, T. (1935) The respiratory quotients of normal and tumour tissue. *Biochem. J.*, **29**, 2433.

HAYNES, M. J. & INCH, W. R. (1976) Effect of metronidazole on the radiocurability and oxygenation of the C3HBA tumor. *Radiat. Res.*, **68**, 184.

IBSEN, K. H. (1961) The Crabtree effect: a review. *Cancer Res.*, **21**, 829.

KIRICUTA, I., MUSTEA, I., ROGOZAN, I. & SIMU, G. (1965) Relations between tumor and metastases. I. Aspects of the Crabtree effect. *Cancer*, **18**, 978.

LEVY, H. B., DAVIDSON, H. M., REINHART, R. W. & SCHADE, A. L. (1953) Metabolism of the DBA mouse ascites thymoma. *Cancer Res.*, **13**, 716.

LOCKER, A. & SPITZY, K. H. (1956) Die Atmung von Zellen und Geweben *in vitro* als physiologische Methode und als Modellvorgang. *Z. Ges. Esper. Med.*, **127**, 1.

LOOMIS, W. F. & LIPMAN, F. (1948) Reversible inhibition of the coupling between phosphorylation and oxidation. *J. Biol. Chem.*, **173**, 807.

MUSTEA, I. (1974) Experimental and clinical considerations concerning the radiotherapeutical exploitation of the Crabtree effect in radiotherapy. *Neoplasma*, **21**, 525.

MUSTEA, I., BARA, A., PETRESCU, I. & REVESZ, L. (1978) Effect of anoxic radiosensitizers on cellular and mitochondrial oxygen consumption and respiration control ratio. *Br. J. Cancer*, **37**, Suppl. III, 159.

MUSTEA, I. & MURESIAN, T. (1967*a*) Study of the Crabtree effect in some bacterial cultures. *Cancer*, **20**, 1499.

MUSTEA, I. & MURESIAN, T. (1967*b*) Contribution a l'interprétation du méchanisme de l'effet Crabtree. *Bull. Soc. Chim. Biol.*, **46**, 43.

NOELL, W. K. (1958) Differentiation, metabolic organization and viability of the visual cell. *Arch. Ophthalmol.*, **60**, 702.

RACKER, E. (1956) Carbohydrate metabolism in ascites tumor cells. *Ann. N.Y. Acad. Sci.*, **63**, 1017.

RAM, D., STETTIGER-KALNER, H. & BLOCH-FRANKENTHAL, L. (1963) Inducers of the Crabtree effect and its release by uncouplers and other agents. *Cancer Res.*, **23**, 600.

WENNER, C. E. & WEINHOUSE, S. (1955) Metabolism of neoplastic tissue. VII. Effects of dinitrophenol and fluoride on glucose oxidation in tumor homogenates. *Cancer Res.*, **15**, 497.

YAMADA, T. (1968) Effects of gamma-irradiation on glycolytic control mechanisms in Ehrlich ascites tumor cells. *J. Radiat. Res.*, **9**, 41.